# Does ICT investment necessarily improve operational performance? An empirical analysis of health services firms in India

**DOI:** 10.1186/s12913-025-12984-3

**Published:** 2025-07-01

**Authors:** Gulam Goush Ansari, Rajorshi Sen Gupta

**Affiliations:** Department of Economics and Finance, Birla Institute of Technology and Science, Pilani, K K Birla Goa Campus, Zuarinagar, Goa 403726 India

**Keywords:** Indian health services firms, ICT investment, Contemporaneous flow of ICT, Stock of ICT, Advertisement and marketing, System GMM

## Abstract

**Background:**

The impact of investing in information and communication technology (ICT) on healthcare sector is widely debated. Specifically, the question of how ICT influences the performance of healthcare firms in developing economies is understudied. This study, therefore, examines the impact of ICT investment on the performance of health services firms in India.

**Methods:**

The system generalized method of moment (GMM) is applied on a longitudinal dataset comprising of 378 firms from 2000 to 2023.

**Results:**

The study presents evidence on differential effect of contemporaneous and accumulated stock of ICT investment on three metrics of firm performance: operating expenses, sales, and profits. There are three key findings of the study. *First*, the accumulated stock of ICT leads to reduced operational costs. In contrast, contemporaneous investment in ICT is found to have an insignificant impact on operating expenses of firms. *Second*, ICT fails to convert the enhanced cost efficiency into higher sales and profits. This result highlights a disconnect between technological advancement and productivity gains. *Third*, advertisement and marketing (A&M) is found to have a significant, positive impact on the sales and profits of health services firms.

**Conclusions:**

The study indicates that health services firms in India are able to utilize ICT to achieve cost efficiency but not strategic positioning in terms of providing differentiated, high-quality healthcare service. Hence, mere ICT investment cannot be considered a unique resource for the firms. It is imperative for health services firms to develop and promote inimitable value propositions. As firms must continuously invest and upgrade ICT to enhance the quality of healthcare services, they should concomitantly spend on advertisement and marketing (A&M) to improve the perception and brand value among existing and potential patrons.

## Introduction

Information technology is being hailed as a key source of revolution in the production processes across industries. Not surprisingly, there has been a growing trend in the use of information and communication technology (ICT) by health services firms, both in advanced and emerging economies. Such an observed trend can be attributed to a substantial reduction in costs of hardware, software, and communication equipment.

Given the increasing demand for affordable and more efficient healthcare services, organizations are keen to adopt technological solutions. ICT has enabled healthcare firms to become more efficient and provide improved quality service [1]. By embracing digital technologies, healthcare organizations are continuously trying to enhance the efficiency of the hospital staff and provide better healthcare services to patients [2].

This study draws motivation from two key intriguing observations. First, healthcare service firms have been investing vigorously in ICT and digital technologies in spite of potentially *negative* returns on investment and various practical problems associated with the adoption of new technologies. Second, notwithstanding the problems associated with ICT, firms are observed to continuously invest in ICT, which increases their *accumulated* stock of technological capability over time. These apparently paradoxical observations are elaborated in the following discussion.

First, health service organizations have been allocating increasing amounts of their budget to the development and maintenance of ICT resources, with the basic objective of improving operational efficiency and patient care. Yet, in spite of the substantial increase in technological investment, organizations have *not* experienced significant improvement in productivity [1, 3]. From the perspective of health service firm managers, it is important to assess the expected return from technological investments when they want to raise capital to expand their business. Hence, it is indeed a matter of concern that there is a potential *negative* return on investment in ICT. The issue becomes even more pertinent in the context of developing countries, where substantial evidence is needed to justify investment in the latest healthcare technologies [4].

In more recent years, hospitals have started integrating artificial intelligence (AI) in diagnostics and treatment plans to provide enhanced quality medical services. The Internet of Things (IoT) is being used in the early diagnosis of diseases, and cloud computing helps doctors in decision-making during exigencies. Likewise, the use of computerized physician order entry (CPOE) is associated with a decrease in medical errors. Additionally, big data analytics is emerging as an instrumental tool in evaluating past disease patterns and predicting future ailments. The endowment of such technologies is expected to reduce the operating expenses of healthcare firms and boost productivity of the hospital staff and medical service providers. Technology-integrated healthcare services significantly enhance the patient experience by providing faster services and personalized care. For instance, telemedicine makes it more convenient for individuals to seek quality medical support irrespective of their geographic location. Hence, ICT can improve clinical and process quality, augmenting a firm’s operational performance [5–7].

Since health services organizations are widely adopting digital technologies, any technological fault can have serious impact on healthcare service. Implementation failure has been noted as a serious problem associated with ICT [1]. There are various anecdotal instances where information technology failures have resulted in disruptions in acute care provision in hospitals [2]. Kim et al. [8] review various problems associated with health information technology. The authors identify critical IT problems due to poor user interfaces, system functionality, and software updates. Such problems have led to delayed healthcare delivery and unfavourable patient outcomes. While health IT involving AI, IoT, and Blockchain has immense potential to transform healthcare services, these technologies also bring important challenges [9]. There are ethical concerns involving the privacy of patients, technological hurdles due to interoperability, and legal complications related to data security [9].

Consequently, it is important to take a cautious approach toward euphoria related to the use of information technology. Extant studies have revealed the potentially *adverse* impact of ICT [10, 11]. Studies have indicated that implementing health management information systems may not lead to efficiency gains [12]. Although ICT has the potential to transform the way economic activities are conducted, its overuse can also negatively affect employee productivity and firm performance. Therefore, it is important for health services firms to carefully re-examine their ICT investment decisions.

Next, we explain the second intriguing observation related to health services firms’ accumulated ICT investment. Investment in ICT has become both a strategic necessity and an operational requirement for firms. In this context, it is important to understand the differential role of *stock* and *flow* of ICT investment. The flow of ICT captures the contemporaneous investment required to augment the existent ICT capabilities. Such investment in ICT ensures that firms remain up-to-date with technological advancements. However, these expenditures alone may not guarantee continual performance improvements without a strong foundation of ICT infrastructure.

According to *the dynamic asset accumulation* model, assets should be accumulated over time to develop a bundle of strategic assets [13]. If a firm does not commit the time and effort to upgrade critical assets, it will experience a loss in competitive advantage. The notion of time compression diseconomies contends that it might be difficult for newcomers to catch up with industry leaders by pursuing a big-bang investment strategy [13]. Firms trying to make sudden and significant technological investments would need more depth of capability. A robust ICT capability is established through persistent learning and accumulation of experience. Therefore, the accumulated *stock* of ICT capital becomes essential for firms to effectively manage their operations, reduce costs, and achieve operational benefits.

It is important to note that although the flow of ICT can be adjusted and replicated rapidly, the stock of ICT capital cannot be modified or replicated easily. Dynamically accumulated ICT stock can offer greater bandwidth to the healthcare firms in terms of implementing more efficient operational practices. Thus, the accumulated stock of ICT is likely to have a more profound and durable impact on firm performance than period-specific investment.

### Gaps and contribution

Given the above discussion on observed trends in ICT investment, it is important to identify the prominent gaps in the existing literature. First, the relationship between ICT investment and firm performance in healthcare is largely focused on developed economies, with limited attention to emerging nations [14–16]. Second, in the Indian context, existing studies predominantly focus on identifying individual-level enablers of technology adoption in healthcare [17–19]. There is scanty literature on firm-level analysis with respect to the impact of ICT investment on healthcare firm performance. Third, there is a dearth of studies investigating the effect of accumulated stock of ICT capital on firm performance. Typically, the literature addresses the business value of the contemporaneous flow of ICT investment at a given point in time [7, 14, 20, 21]. Nonetheless, the outcome of short-term ICT investment need not be realized immediately [22]. In contrast, the accumulated stock of ICT capital is expected to have an impact on organizational learning and operations in a sustained manner. The cumulative ICT investment can thus provide more nuanced insights into technology driven firm performance. The sustained investment in ICT enables firms to overcome complexities associated with technology adoption in the short run.

Given the abovementioned gaps, this study attempts to *contribute* to the literature along the following directions.

First, to the best of the authors’ knowledge, there is a dearth of empirical studies investigating the effect of ICT on the performance of healthcare firms operating in India. By focusing on the Indian healthcare sector, this study addresses the geographical imbalance in the literature, which primarily focuses on the developed economies. The study seeks to provide valuable insights with respect to the efficacy of technology utilization among healthcare firms operating in developing countries like India.

The second novelty of this study is its investigation into the contrasting effects of cumulative stock and flow of ICT investment on healthcare firm performance. While both measures of ICT investment are expected to be important, understanding their potentially differential impacts on firm performance would be essential from an analytical and managerial perspective.

Third, this study examines the impact of ICT investment on the operating expenses, profitability, and sales of health services firms in India. By considering these alternative metrics, the study provides a comprehensive perspective on the role of technology on firm performance.

The remainder of the paper is organized as follows. “Literature review” section discusses the literature and theoretical framework to develop an understanding of the link between ICT and the performance of health services firms. “Data and methodology” section explains the methodology adopted, and econometric results are provided in “Empirical results” section. A critical discussion of the results is presented in “Discussion” section. The implications of the findings of this study for the firm managers are discussed in “Managerial implications” section. “Conclusion” section presents the conclusion of the study, discussing its limitations, followed by suggestions for future research directions.

## Literature review

### Theoretical framework

This study draws on the *resource-based view* (RBV) to understand the link between ICT investment and firm performance in healthcare. The theory postulates that a firm’s unique, non-substitutable, and inimitable resources give it a competitive advantage [23]. Nevertheless, ICT is often perceived as a standardised resource that competitors can easily imitate. Therefore, the success of a firm is contingent not merely on periodic ICT investments but on capabilities developed over time. For instance [24], argues that firms leveraging IT resources to create unique IT capabilities would exhibit superior performance.

A firm’s *stock* of ICT resources that is well aligned with the organizational structure becomes hard to replicate [22, 25, 26]. This depth of integration stimulates synergies between technology and human capital, allowing firms to leverage ICT effectively. In the context of health services firms, a substantial stock of ICT can improve the interoperability of medical data within departments. This, in turn, would support efficient decision-making and better resource allocation. The stock of ICT capital enables institutes to process patient information proficiently, deliver better medical care, and standardise medical records [3, 14]. These capabilities derived from a strong, accumulated ICT base become core competencies that improve the delivery of healthcare services. Such technology-enabled core competencies would also be hard for rival firms to replicate.

A firm with a large stock of ICT resources may also develop enhanced capabilities for innovative activities. In a rapidly changing technological landscape, firms with existing robust ICT capabilities can more readily incorporate new technologies with minimal complexities [27]. This adaptability gives firms a competitive advantage, as they can augment their ICT capabilities to respond to market changes more effectively.

In contrast to the accumulated stock of ICT, the *flow* of ICT investment becomes relevant to accommodate immediate, period-specific operational needs. However, firms’ adjustment to new technology must match the speed at which it flows [28, 29]. In other words, technology may fail to embed quickly into the firm’s existing organizational structure. Although the flow of ICT investment is likely to provide transitory benefits, it may not yield the strategic impact resulting from the accumulated stock of ICT.

Drawing on the RBV, this study seeks to analyse whether the stock of ICT investment can be considered a strategic asset for health services firms. With a large stock of ICT, firms are likely to be better positioned to enhance operational efficiencies and maintain competitive advantage. In contrast, ICT flow is expected to offer limited benefits to the firms. Thus, a firm’s strategy of developing ICT stock aligns with RBV theory, as it contributes to developing valuable, rare, and difficult-to-imitate resources.

### Review of literature and hypothesis development

#### ICT investment and firm performance

Health services firms that effectively leverage their ICT capabilities can provide superior quality healthcare services, reduce medical errors, and augment productivity, thereby achieving a sustainable competitive advantage [7, 15, 30]. Nonetheless, the empirical evidence on the association between ICT and hospital performance is characterised by mixed results [31–33]. This section provides an overview of the literature on the relationship between ICT investment and healthcare firm performance.

##### Linking ICT investment with cost performance

The flow of ICT investment captures a firm’s strategic approach to utilize the latest technology for improved performance. However, its effective application would require regular training programs, which might increase its operating expenses. Cost efficiency is achieved only after the firm becomes more proficient in using the new technologies. Borzekowski [28] observed that ICT usage is associated with decreased costs after three to five years of adoption. Hence, firms with a strong ICT system may experience better capacity utilization. This can effectively reduce overall expenses. For instance, ICT enabled automation has the potential to reduce labor costs and allow hospitals to allocate personnel for higher value-added activities. Within the framework of *transaction cost economics perspective*, ICT can play a crucial role minimizing ex-ante and ex-post transaction costs in hospitals. One of the major ex-ante transaction costs involves the time and resources spent searching for patients past medical records and disseminating that information across various departments. Diffusion of technologies like electronic health records (EHR), radio frequency identification (RFID), and electronic data interface (EDI) improves storage, retrieval, and sharing of medical records, which makes hospitals more cost efficient. Das et al. [3] argued that consistent ICT investment helps hospitals achieve cost efficiency, which appears to persist in the long term. Additionally [31], observed that ease of information sharing due to higher investment in electronic health records (EHR) translates into lower hospital costs. Improved data sharing and coordination among various departments increase the quality of care and eliminate the need for unnecessary tests and readmissions. Likewise [34], had similar findings in their study. Hospitals capitalising bundle of radio frequency identification (RFID) and electronic data interface (EDI) experience enhanced supply cost efficiency, lower personnel cost, and a consistent decrease in readmission rates. These benefits accrue from the persistent use of technology. Therefore, it is hypothesized that:

- H1a: ICT investment is likely to decrease health services firm’s operating expenses.

##### Linking ICT investment with profitability

Investment in ICT has the potential to make healthcare firms more profitable by enhancing efficacy, delivering quality services, and fostering innovation. Unlike the period-specific flow of ICT investment, the cumulative ICT investment would enable the firm to attain an enduring competitive advantage, which aligns with the *resource-based view*. The fundamental premise of this perspective is that a firm’s success is determined by its unique resources and capabilities [23]. Robust ICT capabilities developed over the years are more likely to enable firms to demonstrate operational superiority and better performance.

In the context of U.S., it has been found that hospitals embedding IT in their operations became profitable in the long run [14]. Studies in the subsequent years corroborate the idea that healthcare firms with superior IT orientation reap greater financial benefits [35–41]. It is therefore hypothesized that:

- H1b: ICT investment is expected to have a positive effect on the firm’s profitability.

##### Linking ICT investment with sales

ICT investment by firms is expected to have a favourable impact on their operational cost. Firms can leverage this cost efficiency to pursue a competitive pricing strategy, thereby boosting sales. Moreover, ICT investment would also enable firms to offer personalized healthcare services and specialized treatments. The enhanced service offerings would attract more patients and bolster sales revenue [20, 32, 35]. More recently, firms are embracing digital solutions to provide improved healthcare services. Standardized patient data through EHR has made healthcare delivery faster and more convenient [31]. As a result, firms can cater to a larger base of patients more efficiently. Additionally, the use of digital platforms to manage patient relationships can possibly foster stronger ties with the patients. ICT thus helps firms understand patient needs and provide additional value through wellness programs or follow-up care over the long term. Such strategies can directly increase sales per patient by providing customized services that patients are more likely to value. Therefore, in light of the above discussion, the following hypothesis is formulated.

- H1c: ICT investment is likely to have a favorable impact on sales of health services firms.

##### Advertising and Marketing (A&M)

In addition to investment in technology, advertising and marketing expenditures have also become a strategic necessity for the firms. A firm pursuing a more differentiated marketing approach is likely to perform better [42]. Firms can effectively develop and maintain long-term customer relationships using conventional and modern marketing channels. Digital platforms have recently provided firms with a cost-effective approach to reach out to more customers. Hence, technology enabled marketing strategies have the potential to assist firms in maximizing their revenue and becoming more profitable [43]. Hence, it is hypothesized that,

- H2: Advertising and marketing expenses are expected to have a positive effect on sales and profits of health service firms.

#### Control variables

##### Size

Bigger firms are usually endowed with a more extensive ICT capital base and advanced technology. Hence, larger firms are expected to be more efficient. Hah and Bharadwaj [44] found that hospital size is a crucial determinant of hospital productivity and revenue for hospitals in the U.S. Similarly [16], found that large sized hospitals demonstrate better financial performance.

Nevertheless, another strand of literature suggests an ambiguous relationship between firm size and performance. For instance [45], reports a negative effect of hospital size on its performance. As firm size increases, it might lead to operational and cost inefficiencies, resistance to technological change, and inconsistency in care delivery. Likewise [32], postulates that as firm size increases, there can be *diseconomies of scale* on account of lower information transparency and increased transaction costs. A larger firm is typically characterized by a complex organizational structure. It employs a larger workforce for effective management of operations, leading to increased overhead expenses. Thus, increased operational complexity leads to higher costs for larger firms. Consequently, it is hypothesized that

- H3: Firm size is expected to have an ambiguous impact on operating costs but a favorable impact on sales and profitability of the firms.

##### Firm age

With increasing number of years of operation, firms benefit from their accumulated knowledge and expertise through *learning by doing*. Older firms with greater patient volume, reputation, and financial stability are likely to perform better. For instance [44], found that the older the hospital, the higher the productivity and financial performance. However, as firms grow older, they might become less receptive to innovative ideas due to organizational rigidities. The increased rigidity makes older firms less efficient, resulting in higher expenses and reduced profitability. In contrast, new firms might be more agile and flexible, making them more competent. Kohli et al. [37] observed that the market value of young hospitals is greater than that of their older counterparts. The endowment of the latest technologies and efficient utilization of resources gave young hospitals an edge over their rivals. Hence, in light of this discussion, it is hypothesized that

- H4: As firm age increases, it is expected to reduce operational costs, but increase sales and profits.

##### Location

Healthcare firms in urban areas typically benefit from easy access to slack resources, better infrastructure, and availability of skilled caregivers. Bardhan and Thouin [21] argue that the availability of better healthcare infrastructure has rendered urban hospitals more proficient. They further observed that urban hospitals are more IT-intensive. Hence, a higher degree of IT integration reduces hospital costs and improves operational efficiency. Additionally, serving the affluent class of clients in urban areas also boosts the profitability of firms [41]. Therefore, it is hypothesized that

- H5: Firms located in urban areas are likely are expected to have lower operational costs, but higher sales and profits than the firms located in rural areas.

##### Ownership

Firm ownership structure has been widely studied as a critical determinant of organizational performance across industries, including healthcare [41, 44, 46]. Specifically, public and private ownership structures exhibit varying features that can affect operational efficiency and firm performance differently.

Public limited firms are often characterized by easier access to equity and lesser capital constraints [47]. However, the quest to balance profit maximising goals and social objectives sometimes makes them less profitable. In contrast, private limited firms are primarily profit-oriented entities. In the context of the Indian healthcare sector, the effect of ownership structure on firm performance is important. Public healthcare firms often face operational challenges due to bureaucratic inefficiencies. A broader mandate to provide more affordable healthcare services often limits their ability to generate profits. Conversely, private firms typically operate in competitive urban markets with a higher proportion of relatively affluent patients. These firms are able to leverage advanced technologies to enhance profitability and sales. Hence, in light of this discussion, it is hypothesized that

- H6: Public firms are likely to exhibit higher costs, lower sales, and profitability than private firms.

## Data and methodology

### Sample description

The data has been extracted from the Centre for Monitoring Indian Economy (CMIE) Prowess database from 2000 to 2023 for 378 healthcare firms. Operating expenses, profitability, and sales are used as alternative performance indicators for firms. A concise description of the variables used in the study is provided in Table 1. In the present study, the term *health services firms* refers to entities classified in the CMIE Prowess database under the following categories: (1) hospitals and healthcare centers, (2) medical and health services, and (3) medical testing centers and laboratories. The total population of healthcare firms in the target cohort is 2505. The study sample was selected based on the availability of consistent and complete data for key variables essential to our analysis. For instance, a significant number of firms did not report data on the key variables like ICT investment and A&M expenses across the study period, thereby making them unsuitable for inclusion in the sample. Additionally, as per the OECD, ICT investment is defined as the acquisition and utilization of equipment and software used in the production process for a period exceeding one year. Therefore, the firms reporting data on ICT investment for more than one year are considered for this study. This led to the final study sample of 378 firms.

**Table 1:** Variable description

Variable	Notation	Measure	Literature citation
*Dependent variable*			
Operating expenses	lnOE	Operating expenses = Natural logarithm (doctors/consultants fees + expenses on medical consumables + other miscellaneous expenses of hospitals) [Rupees in Million]	[3, 21, 32]
Profit before depreciation, interest, tax and amortization	lnPBDITA	PBDITA = Natural logarithm of [Profit after tax + write offs + (total provisions – provision for obsolescence of raw material – Provision for estimated losses on onerous contracts) + total tax expenses + amortisation + depreciation + financial services expenses] [Rupees in Million]	[15, 33]
Sales	lnSales	Natural log of sales [Rupees in Million]	[36]
*Explanatory variables*			
ICT	lnICT	Natural logarithm of net expenses towards software, net expenditure on computers and IT systems and communication equipment [Rupees in Million]	[48]
Cumulative ICT	lnCum_ICT	$\:\text{ln}\left(\sum\:_{2000}^{t}I{CT}_{t}\right),\:t=2000,\dots\:,2023$. [Rupees in Million]	[48]
Size	lnAssets	Natural logarithm of total assets [Rupees in Million]	[5, 32]
Age	lnAge	Natural logarithm of Age = Natural logarithm (Year of observation-Year of incorporation)	[7, 35, 44]
Advertising and Marketing expenses	lnA&M	Natural log of (Marketing expenses + Advertising expenses) [Rupees in Million]	[42, 43]
Location	Location	= 1 if firm is located in urban area, 0 otherwise	[5, 31, 45]
Ownership	Ownership	= 1 if firm is public limited, 0 otherwise	[41, 44]

### Econometric specification

It is understood that the relationship between ICT and firm performance in healthcare is likely to be subject to potential endogeneity [49]. The non-random nature of ICT investment implies that firms do not make investments in ICT arbitrarily. Instead, investment decisions are driven by market conditions, technological advancements, and organizational requirements.

To address the potential endogeneity problems, the System GMM technique is deemed appropriate. The method was developed by [50–52]. The econometric model is specified as follows.

1 $$\:{Y}_{it}=\alpha\:{Y}_{i,t-1}+\beta\:{X}_{it}{+\gamma\:{Z}_{it}+\epsilon\:}_{it}$$

Where $\:{Y}_{it}$ denotes the dependent variable. $\:{Y}_{it-1}$ is the lagged dependent variable. $\:{X}_{it}$ is ICT or cumulative ICT, depending on which specification is being estimated. $\:{Z}_{it}$ denotes the control variables and $\:{\epsilon\:}_{it}$ is the disturbance term that has two orthogonal components: the unobserved individual-specific effect, $\:{\mu\:}_{i}$, and the idiosyncratic error term, $\:{\vartheta\:}_{it}$, such that-

$$\:{\epsilon\:}_{it}={\mu\:}_{i}+{\vartheta\:}_{it}$$

The lagged dependent variable $\:{Y}_{i,t-1}$ in Eq. 1 is correlated with the unobserved firm-specific effect $\:{\mu\:}_{i}$, which gives rise to dynamic panel bias [53]. There are two approaches to address this endogeneity. The first, central to the ‘Difference GMM’ estimator, is to eliminate the fixed effects through data first differencing Eq. 1 as follows-

2 $$\:{\varDelta\:Y}_{it}=\alpha\:{\varDelta\:Y}_{i,t-1}+\beta\:{\varDelta\:X}_{it}+{\gamma\:{\Delta\:}{Z}_{it}+\varDelta\:\vartheta\:}_{it}$$

Though differencing eliminates the fixed effects i.e. $\:{\mu\:}_{i}$, endogeneity in the Eq. 2 persists, because the lagged dependent variable $\:{Y}_{i,t-1}$ in $\:{\varDelta\:Y}_{i,t-1}={Y}_{i,t-1}-{Y}_{i,t-2}$ correlates with the idiosyncratic error term $\:{\vartheta\:}_{i,t-1}$ in $\:{\varDelta\:\vartheta\:}_{it}={\vartheta\:}_{it}-{\vartheta\:}_{i,t-1}$.

Thus, in the second approach the remedial measure is to instrument the lagged dependent variable $\:{Y}_{i,t-1}$ and any other potentially endogenous variables using instruments that are uncorrelated with the fixed effects. The differenced and lagged values fulfil the exogeneity conditions and hence act as valid instrumental variables [54].

The system GMM estimator stacks both differenced and level equations to improve efficiency and mitigate endogeneity bias by generating internal instruments [53, 55].

$$\begin{aligned} \begin{bmatrix}Y_{it}\\\varDelta Y_{it}\end{bmatrix}=\alpha\begin{bmatrix}Y_{it-1}\\\varDelta Y_{it-1}\end{bmatrix}+\beta\begin{bmatrix}X_{it}\\\varDelta X_{it}\end{bmatrix}+\gamma\begin{bmatrix}Z_{it}\\\varDelta Z_{it}\end{bmatrix}+\vartheta_{it} \end{aligned}$$

The regressions were estimated using STATA 17, by applying the *xtabond2* command with *collapse* option to limit instrument proliferation [53]. Since the two-step standard errors can be downward biased, the Windmeijer corrected standard errors is used for more robust results [53].

To confirm for the robustness of the results, the Arellano-Bond test of first and second-order serial correlation and the Hansen test of overidentification were used. According to [50], the GMM estimator requires the presence of first-order serial correlation but no second-order serial correlation in the residual. For a robust result, we ought to reject the null hypothesis of no first-order serial correlation in the residual at 5% level of significance. In the case of the second-order serial correlation, the null hypothesis of no serial correlation should be accepted at 10% significance. In addition, the Hansen test of overidentification restrictions checks for the validity of instruments. Finally, the number of instruments in the results should not exceed the number of groups. All of these criteria are satisfied in the ensuing regressions, which establishes that the results are robust.

### Descriptive statistics

Table 2 presents the summary statistics of the variables used in the study. The mean values of ICT and cumulative ICT suggest that the flow and stock of ICT are at moderate levels, in comparison to sales or assets. This indicates that ICT may not be deeply embedded in the operations of Indian healthcare firms. Likewise, the mean value of A&M expenses also indicates that firms are not using aggressive marketing and sales strategies. Nearly 96% of firms are located in urban areas, and almost 47% are publicly owned.

**Table 2:** Descriptive statistics

Variable	Obs	Mean	Std. Dev.	Min	Max	Skewness	Kurtosis	Jarque-Bera	Probability
OE	3248	560.35	1702.39	−1.2	26,946	7.73	82.43	886242.9	0.0000
PBDITA	4266	230.83	843.50	−3173.7	18,482	9.83	150.25	3,923,181	0.0000
Sales	4034	1269.49	4148.33	0.1	97,944	11.50	191.62	6,069,129	0.0000
ICT	3861	19.95	70.74	−0.1	1104.6	7.96	82.99	1,070,356	0.0000
Cum_ICT	3862	113.58	421.25	−0.1	7749.6	8.94	106.83	1,786,476	0.0000
A&M	3335	40.41	145.36	0.1	2187.8	8.45	91.70	1,133,191	0.0000
Age	9072	11.68	10.30	0	56	0.72	2.94	798.5071	0.0000
Size	4434	2237.30	7839.91	−0.1	25,082	9.15	110.34	2,190,877	0.0000
Location	9072	0.96	0.17	0	1				
Ownership	9072	0.47	0.49	0	1				

## Empirical results

The regression results with respect to the three alternative performance metrics: operating expenses, profitability, and sales are presented in Tables 3, 4 and 5 respectively. The coefficients of the lagged dependent variables are highly significant across all models. This affirms the application of dynamic panel data analysis. Based on the results in Tables 3, 4 and 5, it is evident that there is a first-order serial correlation since the null hypothesis of no first-order serial correlation is rejected, i.e., AR(1) values being significant at a 5% level of significance. Likewise, the null hypothesis of no second-order serial correlation is accepted because AR(2) values are not statistically significant at a 10% level of significance. Hansen test values being insignificant at 10% indicates that the instruments used are valid. Additionally, number of instruments are less than the number of firms in the study. Hence, the application of two-step system GMM leads to robust results.

**Table 3:** Impact of ICT investment on operating expenses

Variables	(1)	(2)	(3)	(4)	(5)	(6)
	lnOE	lnOE	lnOE	lnOE	lnOE	lnOE
L.lnOE	0.536^***^	0.527^***^	0.527^***^	0.616^***^	0.608^***^	0.608^***^
	(0.0944)	(0.0931)	(0.0928)	(0.0978)	(0.0978)	(0.0979)
lnICT	0.139	0.133	0.121			
	(0.133)	(0.133)	(0.137)			
lnCum_ICT				−0.328^***^	−0.323^***^	−0.317^***^
				(0.119)	(0.120)	(0.118)
lnAge	−0.0614	−0.0509	−0.0404	−0.0149	−0.00901	−0.0188
	(0.0925)	(0.0891)	(0.0818)	(0.0848)	(0.0831)	(0.0764)
Size	0.374^**^	0.382^**^	0.407^**^	0.679^***^	0.677^***^	0.676^***^
	(0.170)	(0.171)	(0.180)	(0.124)	(0.122)	(0.123)
Location		−0.457^***^	−0.448^***^		−0.266	−0.277
		(0.159)	(0.158)		(0.195)	(0.190)
Ownership			−0.113			0.0250
			(0.0854)			(0.0909)
Constant	−0.0693	0.348	0.215	−1.358^***^	−1.081^**^	−1.075^**^
	(0.786)	(0.759)	(0.805)	(0.456)	(0.502)	(0.501)
Observations	2,656	2,656	2,656	2,659	2,659	2,659
Number of Firms	302	302	302	302	302	302
Number of Instruments	39	40	41	39	40	41
Wald Chi2	32737.95	34514.13	36740.48	26870.80	26982.61	27268.03
AR(1) (p-value)	0.001	0.001	0.001	0.001	0.001	0.001
AR(2) (p-value)	0.560	0.568	0.568	0.513	0.518	0.519
Hansen Test (p-value)	0.129	0.149	0.181	0.312	0.327	0.319
Year FE	Yes	Yes	Yes	Yes	Yes	Yes

Standard errors in parentheses

^***^p < 0.01, ^**^p < 0.05, ^*^p < 0.1

**Table 4:** Impact of ICT investment on profitability

Variables	(1)	(2)	(3)	(4)	(5)	(6)
	lnPBDITA	lnPBDITA	lnPBDITA	lnPBDITA	lnPBDITA	lnPBDITA
L.lnPBDITA	0.412^***^	0.412^***^	0.396^***^	0.431^***^	0.430^***^	0.420^***^
	(0.0963)	(0.0962)	(0.0989)	(0.0941)	(0.0942)	(0.0949)
lnICT	−0.0822	−0.0807	−0.0924			
	(0.0986)	(0.0992)	(0.103)			
lnCum_ICT				−0.232^*^	−0.231^*^	−0.226^*^
				(0.140)	(0.140)	(0.136)
lnA&M	0.205^**^	0.207^**^	0.210^**^	0.216^**^	0.218^**^	0.217^**^
	(0.0932)	(0.0935)	(0.0959)	(0.0968)	(0.0971)	(0.0938)
lnAge	−0.0692	−0.0685	−0.0468	0.0447	0.0457	0.0541
	(0.0815)	(0.0811)	(0.0793)	(0.0998)	(0.0995)	(0.0854)
Size	0.547^***^	0.545^***^	0.598^**^	0.613^***^	0.611^***^	0.630^***^
	(0.202)	(0.202)	(0.235)	(0.189)	(0.188)	(0.191)
Location		0.114	0.0996		0.102	0.0974
		(0.166)	(0.194)		(0.185)	(0.195)
Ownership			−0.186			−0.0950
			(0.120)			(0.101)
Constant	−0.908	−1.006	−1.234	−1.041	−1.128	−1.205
	(0.883)	(0.832)	(0.980)	(0.771)	(0.732)	(0.745)
Observations	2,449	2,449	2,449	2,451	2,451	2,451
Number of Firms	298	298	298	298	298	298
Number of Instruments	36	37	38	36	37	38
Wald Chi2	31249.69	31468.08	27143.37	29954.89	29976.93	29667.85
AR(1) (p-value)	0.000	0.000	0.000	0.000	0.000	0.000
AR(2) (p-value)	0.275	0.275	0.311	0.280	0.283	0.302
Hansen Test (p-value)	0.234	0.232	0.291	0.169	0.170	0.195
Year FE	Yes	Yes	Yes	Yes	Yes	Yes

Standard errors in parentheses

^***^p < 0.01, ^**^p < 0.05, ^*^p < 0.1

**Table 5:** Impact of ICT investment on sales

Variables	(1)	(2)	(3)	(4)	(5)	(6)
	lnSales	lnSales	lnSales	lnSales	lnSales	lnSales
L.lnSales	0.418^***^	0.415^***^	0.412^***^	0.471^***^	0.468^***^	0.472^***^
	(0.0687)	(0.0680)	(0.0678)	(0.0741)	(0.0736)	(0.0738)
lnICT	−0.100	−0.0989	−0.0937			
	(0.0845)	(0.0828)	(0.0799)			
lnCum_ICT				−0.163^**^	−0.161^**^	−0.165^***^
				(0.0645)	(0.0646)	(0.0638)
lnA&M	0.0482	0.0504	0.0604	0.0913^*^	0.0903^*^	0.0988^**^
	(0.0577)	(0.0563)	(0.0556)	(0.0508)	(0.0502)	(0.0478)
lnAge	−0.0992	−0.0901	−0.0526	0.0393	0.0405	0.0584
	(0.0910)	(0.0869)	(0.0788)	(0.0663)	(0.0655)	(0.0608)
Size	0.710^***^	0.701^***^	0.688^***^	0.573^***^	0.572^***^	0.553^***^
	(0.162)	(0.158)	(0.146)	(0.104)	(0.103)	(0.0950)
Location		−0.245	−0.223		−0.145	−0.131
		(0.172)	(0.170)		(0.127)	(0.125)
Ownership			−0.162^*^			−0.0500
			(0.0852)			(0.0684)
Constant	−0.634	−0.344	−0.316	−0.156	−0.00234	0.0640
	(0.683)	(0.633)	(0.591)	(0.406)	(0.406)	(0.380)
Observations	2,880	2,880	2,880	2,883	2,883	2,883
Number of Firms	319	319	319	319	319	319
Number of Instruments	36	37	38	36	37	38
Wald Chi2	53989.72	54846.49	55448.88	79638.97	80522.61	80980.24
AR(1) (*p*-value)	0.023	0.023	0.023	0.018	0.018	0.018
AR(2) (*p*-value)	0.300	0.298	0.297	0.255	0.253	0.255
Hansen Test (*p*-value)	0.397	0.403	0.458	0.320	0.328	0.313
Year FE	Yes	Yes	Yes	Yes	Yes	Yes

Standard errors in parentheses

^***^p < 0.01, ^**^p < 0.05, ^*^p < 0.1

The results reported in Table 3 indicate that there is indeed a differential effect of flow and stock of ICT on the operating expenses of health services firms. The flow of ICT has a positive, insignificant impact on operating expenses, as reported in specifications 1–3. However, in specifications 4–6, cumulative ICT’s effect is negative and significant on operating expenses. This result indicates that the stock of ICT capital enables firms to become *cost-efficient*. Thus, H1a is supported, specifically for the cumulative stock of ICT investment.

With respect to hypotheses H1b and H1c, cumulative ICT is found to have a negative and significant impact on profitability and sales, as shown in Tables 4 and 5. Likewise, the flow of ICT investment negatively affects both profitability and sales, although the effects are not significant. Hence, these results do not support H1b and H1c.

Table 3 shows that operating expenses would increase as firm size increases. This implies that large firms tend to be *less* cost-efficient than smaller firms, which indicates possible diseconomies of scale effect. Nonetheless, firm size positively affects profitability and sales in Tables 4 and 5. The larger firms benefit from access to slack resources and a larger capital base. Better resource endowment enables such firms to invest in the latest technologies and pursue aggressive marketing strategies, thereby increasing sales and profits. Hypothesis H3 is thus supported for all of the performance metrics.

With respect to hypothesis H4, as firm age increases, there is a reduction in operational costs, although the result is statistically insignificant. Moreover, firm age has a positive but insignificant effect on profitability and sales in specifications (4) to (6) in Tables 4 and 5.

Regarding Hypothesis H5, firms located in urban areas are more cost efficient than those in rural areas. The result is significant in specifications 2 and 3 in Table 3. In terms of profitability (Table 4), urban firms are found to be performing better than rural firms, although the result is not statistically significant. In terms of sales (Table 5), surprisingly, urban firms perform worse than rural firms, although the result is insignificant.

With respect to Hypothesis H6, there is an ambiguous impact of ownership structure on operational costs. As hypothesized, in terms of both profits and sales, the public firms are found to be worse than the private firms. However, the results are not statistically significant.

The impact of A&M expenses is significant and positive on both the profitability and sales of firms in Tables 4 and 5. Thus, investment in marketing and sales would lead to better firm performance. Consequently, hypothesis H2 is supported unambiguously.

## Discussion

Given the results of our analysis, we are now able to address the major question: does ICT investment necessarily affect the performance of the health services firms? It is found that the cumulative stock of ICT, not the contemporaneous ICT investment, makes firms cost-efficient. Results indicate a statistically insignificant impact of contemporaneous ICT investment on the operating expenses of health services firms. This can be attributed to a lengthy learning period and adjustment costs associated with technology usage [28, 29]. In contrast, the significant, *negative* association between operating expenses and cumulative ICT indicates that the firms are able to effectively integrate ICT into their operational processes and exhibit cost efficiency in the long run [28].

The improved decision-making and resource optimization due to increased ICT capabilities make firms more cost-efficient [21, 28, 31]. The automation and digitization of processes mitigate human error in patient data management and inventory control. The favourable impact of ICT on operating expenses can attributed to the *transaction cost economics* theory [56]. The accumulation of technology facilitates the automation of processes, improves information flow between different departments, and enhances the coordination of tasks, thereby leading to lower operating costs. Consistent technological accumulation can also encourage firms to implement innovative business models that lead to efficiencies and reduced costs.

Though the cumulative stock of ICT investment enables firms to attain cost efficiency, this efficiency does not translate into increased sales and profitability. The results suggest that both contemporaneous and stock of ICT investments fail to increase profit and sales. This indicates that health services firms in India are unable to convert their operational efficiencies to higher sales and profits. The literature attributes this phenomenon to *the productivity paradox* of information technology. Solow succinctly pointed out the contradictory impact of ICT investment on firm productivity. Extant studies also highlight this paradox in healthcare sector [33, 57, 58].

In light of this finding, it is essential to identify the plausible reasons for this paradoxical relationship between ICT investment and firm profits or sales. One of the fundamental reasons could be the rigidity of the ICT legacy system of health services firms in India. Studies indicate an overdependence of Indian healthcare on conventional technologies [18, 59, 60]. The penetration of cutting-edge technologies in India is extremely low [59, 61], and only a few hospital chains invest in them [18, 62]. Moreover, overdependence on outdated technologies results in higher switching costs when adopting new technologies [63]. The rigidity of the ICT legacy system can thus limit firms’ ability to adapt to the changing business requirements. Organizational inertia, lack of technical expertise, and high cost of adoption are potential challenges that restrict the integration of new technologies with the firm’s existing legacy system. There is evidence of resistance from caregivers to adopting new technologies [64, 65]. Integration of new technologies necessitates a change in organizational structure and policies. Indian healthcare firms, being laggards in technology usage, might still lack sufficient skills to apply cutting-edge technologies like blockchain, AI, and HER [61, 66]. Hence, Indian healthcare professionals tend to demonstrate lower digital readiness vis-à-vis their global counterparts. Additionally, the high cost associated with new technology can make its integration difficult and result in firms’ reluctance to use it [65].

The underutilization of ICT capabilities has also adversely affected the performance of the Indian healthcare sector. Most health records are paper-based, reducing patient records’ interoperability with other departments or hospitals [60]. The firms are unable to provide better healthcare because updated patient health records are often unavailable. When patients are referred to the other hospital for specialised treatment, the patient record remains with the same hospital [19, 60, 62]. This lack of interoperability makes firms less proficient in providing quality care to patients.

The adverse impact of ICT on profitability and sales may also be attributed to the nature of ICT investment. The firms may use ICT to address operational and administrative issues rather than strategic expansion activities [67]. It is plausible that Indian health services firms primarily focus on automating and digitizing *administrative* processes rather than enhancing patient service. However, a firm’s superior performance is determined by operational excellence and strategic positioning in the market [68]. Better cost efficiency indicates that firms predominantly invest in *transactional* and *informational* ICT applications. Aral and Weill [69] argue that these strategies lead to cost reduction for firms. The authors point out that transactional investment automates back-office activities and minimizes costs. Informational transactions facilitate a smooth flow of information within the departments, which minimizes human error in patient data management. Tallon et al. [70] found that firms whose IT strategy is aligned with operational efficiency or strategic positioning tend to realize lower returns. An effective amalgamation of *both* strategies can be more profitable for firms. In light of the results obtained in this study, it may be argued that health services firms in India are able to use ICT to achieve operational efficiency but not strategic positioning in terms of providing differentiated, high-quality healthcare service. Therefore, firms ought to strategize on service diversification and more extensive customer outreach.

The results of this paper suggest that customer outreach initiatives through higher A&M expenses are expected to produce improved sales and profitability. Given India’s large patient volume and varying willingness to pay for healthcare services, a differential pricing strategy should be appropriately designed. Additionally, A&M expenses enable a firm to communicate the unique attributes of its healthcare services to create a brand recall effect, thereby fostering competitive advantage.

## Managerial implications

The critical question remains: how can technology lead to better healthcare service and improved financial performance of healthcare service providers? What could be some of the possible ways of utilizing ICT to improve the profitability of healthcare firms? In a developing country like India, with approximately 7.5 doctors per 10,000 people, there is immense scope for digital technologies toward the provision of affordable and accessible healthcare. Undoubtedly, investment in technology is necessary to address the challenges faced by healthcare sector. For instance, Clinical Decision Support System (CDSS) can be used to prescribe evidence-based medicine. Nonetheless, effective deployment of such technologies would critically depend on access to clean, accurate, and consistent historical medical data related to patients. Only when such data are available, healthcare service providers would be able to make better decisions with lesser wait times. Therefore, targeted investment is needed toward the development of technological solutions that can empower healthcare service providers. This would lead to greater efficiency of physicians, better quality of healthcare services, and consequently improved financial outcomes for the organizations.

Digital healthcare solutions require well-established infrastructure involving electronic health records, AI-driven diagnostics, and treatment involving IoT and robotics. The creation of such digital healthcare infrastructure would need massive, coordinated investment from both the government and private sector.

The findings of this study have several important implications for health services firm management. One of the striking results indicates that operational performance, especially sales and profitability, is not solely contingent on ICT investment. Therefore, investment in ICT is a necessary but *not* sufficient condition for a firm to attain better performance [24, 26]. Proper alignment of ICT with business strategy is essential for the firm to emerge as an entity with superior operational performance [22, 71]. Additionally, the synergy of ICT with organizational structure plays an instrumental role in realising the potential benefits of technology. Agha [57] argue that the returns of ICT investment might not be realised without changes in the current organizational structure. The result that ICT fails to improve the financial performance of health services firms might indicate firms’ inability to align ICT with their core business processes.

In the context of health services firms in India, there has been organizational inertia to change the legacy technologies. This may be attributed to budgetary constraints and a lack of technical expertise to adopt new technologies. Indian healthcare professionals might be less proficient in adopting ICT [61, 66]. Hence, it is necessary to train healthcare professionals to assimilate the latest technologies with the least resistance. Without such concerted effort, health services firms would be saddled with outdated technologies. This would reduce their repositioning ability in an evolving business landscape [72]. Hence, as the existing bundle of ICT capital depreciates, it becomes imperative for firms to replenish them with investment in new technologies.

Strategic investment in ICT might lead to product and process innovation for health services firms, albeit in the short run. Eventually, such technologies would become easily accessible across competing firms. Therefore, the benefits of strategic investment in ICT may fade away as all competing firms reach a similar level of ICT usage. For instance [69], observed that strategic investment in IT has a significant bearing on service delivery. But when universally adopted, the investment in IT becomes non-strategic. The use of similar forms of ICT brings technological uniformity to competing firms. Thus, a firm would be too naïve to expect a sustainable competitive advantage from ICT investment alone.

The above discussion suggests that mere ICT investment can no longer be considered a unique resource. Consequently, it becomes imperative for firms to develop inimitable value propositions. This is where the importance of the A&M strategy emerges. In light of the econometric results obtained in this study, expenses on A&M can be a significant factor in improving the sales and profitability of healthcare firms. As firms must continuously invest and upgrade ICT to enhance the *quality* of healthcare services, they should simultaneously spend on A&M to improve the perception and brand value among existing and potential patrons. Moreover, an effective synergy of ICT and marketing capabilities can enable firms to transform operational efficiencies into market responsiveness, thereby creating differentiated values for a sustainable competitive advantage.

## Conclusion

This study provides valuable insights into the impact of ICT investment on the performance of health services firms in India. This study has three core conclusions. First, it is found that firms with *accumulated* stock of ICT are able to achieve cost efficiency. Therefore, firms ought to be persistent in order to reap the operational cost benefits from ICT investment. Second, the firms are unable to utilize ICT to improve their sales and profitability. This suggests the presence of ICT productivity paradox in the Indian healthcare sector. Third, the strategic role of marketing is established by the positive effect of A&M on the sales and profitability of the firms. Consequently, firms must continually invest in ICT to enhance healthcare service quality and spend on A&M to improve brand perception among existing and potential patrons.

The study acknowledges several limitations, which warrant further consideration for future research.

First, given the available data, this study focuses primarily on traditional ICT components, such as hardware and software. Overdependence of a firm’s ICT legacy system on such components fails to encompass the latest advancements in the ICT landscape. Emerging technologies like AI, blockchain technology, EHR, and telemedicine are gaining traction in healthcare. These cutting-edge technologies hold significant potential to revolutionize firm performance. Future research should probe into the impact of these technologies on health services firm performance.

Second, given the challenges related to inefficient management of medical records, cybersecurity, and privacy concerns in Indian healthcare, the role of robust digital health policies becomes crucial. The effective formulation and implementation of such policies can assist healthcare institutions in harnessing the full potential of digital health measures. Digital health policies are vital for strengthening healthcare systems and enhancing the quality of services. They minimize healthcare delivery costs, streamline medical processes, and facilitate more cost-effective digital consultations. Future research is warranted to examine the effectiveness of digital health policies in improving institutional performance.

Third [10], used a mix of ICT capital and ICT labour as a measure of a firm’s ICT capability. This approach provides a comprehensive understanding of a firm’s ability to leverage ICT capabilities. In contrast, this study considers the stock of ICT capital accumulated over time. Though ICT capital provides a strong technological foundation, its effectiveness depends on the skills and expertise of the labour who are actually using the technology. Hence, firms endowed with a large amount of ICT capital but insufficient skilled labor might underutilize the technology. This, in turn, would lead to suboptimal output and profits. Subject to available data, future research may consider using ICT capital along with the labor to understand the impact of technology on firm performance.

The fourth limitation of this study is related to the classification of health services firms. The analysis relies on data obtained from the Prowess database, which categorizes entities under broad headings such as hospitals and healthcare centres, medical and health services, and medical testing centres and laboratories. However, the database does not provide information on the profit orientation (for-profit versus non-profit) of these firms. As a result, the study is unable to distinguish explicitly between for-profit, non-profit institutions, which may differ significantly in their operational structures and efficiency outcomes.

Finally, the study evaluates firm performance by using metrics like operating expenses, profitability, and sales. While these indicators are critical, they may not capture the broader dimensions of performance relevant to healthcare firms. Metrics such as quality of care, patient safety, and patient satisfaction might provide a more holistic view of the impact of ICT on firm performance. Incorporating these non-financial performance indicators in future research would help stakeholders understand the multidimensional effects of ICT investments.

## Data Availability

The data were collected from CMIE Prowess database available from https://prowess.cmie.com/.

## Abbreviations

ICT: Information and communication technology
GMM: Generalized method of moment
A&M: Advertisement and marketing
AI: Artificial intelligence
IoT: Internet of things
CPOE: Computerized physician order entry
RBV: Resource-based view
EHR: Electronic health records
RFID: Radio frequency identification
EDI: Electronic data interface
CMIE: Centre for monitoring Indian economy
OE: Operating expenses
PBDITA: Profit before depreciation, interest, tax and amortization
FE: Fixed effect
CDSS: Clinical Decision Support System
